# Design, Analysis, and Development of Low-Cost State-of-the-Art Magnetorheological-Based Microprocessor Prosthetic Knee

**DOI:** 10.3390/s24010255

**Published:** 2024-01-01

**Authors:** Muhammad Usman Qadir, Izhar Ul Haq, Muhammad Awais Khan, Kamran Shah, Houssam Chouikhi, Mohamed A. Ismail

**Affiliations:** 1 Advanced Robotics & Automation Lab, Department of Mechatronics Engineering, University of Engineering & Technology, Peshawar 25000, Pakistan; usmanqadir91@uetpeshawar.edu.pk (M.U.Q.); mawaiskhan0486@gmail.com (M.A.K.); 2 Department of Mechanical Engineering, King Faisal University, Hofuf Al Ahsa 31982, Saudi Arabia; hchouikhi@kfu.edu.sa (H.C.); maismail@kfu.edu.sa (M.A.I.); 3 Laboratory of Electromechanical Systems (LASEM), National School of Engineers of Sfax, University of Sfax, Sfax 3038, Tunisia

**Keywords:** transfemoral amputation, semi-active prosthetic knee, magnetorheological damper, hierarchical control architecture, variable current controller

## Abstract

For amputees, amputation is a devastating experience. Transfemoral amputees require an artificial lower limb prosthesis as a replacement for regaining their gait functions after amputation. Microprocessor-based transfemoral prosthesis has gained significant importance in the last two decades for the rehabilitation of lower limb amputees by assisting them in performing activities of daily living. Commercially available microprocessor-based knee joints have the needed features but are costly, making them beyond the reach of most amputees. The excessive cost of these devices can be attributed to custom sensing and actuating mechanisms, which require significant development cost, making them beyond the reach of most amputees. This research contributes to developing a cost-effective microprocessor-based transfemoral prosthesis by integrating off-the-shelf sensing and actuating mechanisms. Accordingly, a three-level control architecture consisting of top, middle, and low-level controllers was developed for the proposed prosthesis. The top-level controller is responsible for identifying the amputee intent and mode of activity. The mid-level controller determines distinct phases in the activity mode, and the low-level controller was designed to modulate the damping across distinct phases. The developed prosthesis was evaluated on unilateral transfemoral amputees. Since off-the-shelf sensors and actuators are used in i-Inspire, various trials were conducted to evaluate the repeatability of the sensory data. Accordingly, the mean coefficients of correlation for knee angle, force, and inclination were computed at slow and medium walking speeds. The obtained values were, respectively, 0.982 and 0.946 for knee angle, 0.942 and 0.928 for knee force, and 0.825 and 0.758 for knee inclination. These results confirmed that the data are highly correlated with minimum covariance. Accordingly, the sensors provide reliable and repeatable data to the controller for mode detection and intent recognition. Furthermore, the knee angles at self-selected walking speeds were recorded, and it was observed that the i-Inspire Knee maintains a maximum flexion angle between 50° and 60°, which is in accordance with state-of-the-art microprocessor-based transfemoral prosthesis.

## 1. Introduction

Lower limb amputation (LLA) is a life-changing event for an amputee, resulting in various physical and psychological complications [1]. Major causes of LLA include road accidents, peripheral vascular diseases, and injuries caused by sharp objects [2]. McDonald et al. reported nearly 35.3 million limb amputees worldwide, with the highest amputation rate in East and South Asia [3]. LLA can be broadly categorized as transfemoral (above-knee) amputation (TFA), transtibial (below-knee) amputation (TTA), and hip/knee/foot disarticulation. The rehabilitation of below-knee amputees, including TTA and foot disarticulation, is uncomplicated due to knee joint preservation during amputation [4]. However, mechanized and intelligent devices are essential for the rehabilitation of above-knee amputees; therefore, transfemoral prostheses (TP) constitute an active area of research.

TP has emerged from mechanical devices to state-of-the-art microprocessor-based prosthetic knee joints (MPKJs). Although mechanical TPs are cost-effective, lightweight, and durable, they lack integrated sensors and control architecture, resulting in an unnatural gait, mental stress, physical fatigue, abnormal heartbeat, high energy expenditure, and inability to traverse uneven terrains/stairs/ramps [5,6]. To address these issues, microprocessor-based prosthetic knee joints (MPKJs) have gained significant importance in the last two decades [7]. MPKJ utilizes data from various sensors to control the instantaneous resistance/assistance of the knee joint to simulate normal biological knee function while the amputee is performing activities of daily living (ADLs) [8,9]. The advantages of MPKJs over mechanical TPs include superior balance, an increased awareness of amputee about the position of the prosthesis in real time, the ability to climb stairs, walk on ramps, less concentration on the mechanics of walking, and lower energy expenditure while performing ADLs [10,11].

MPKJs, based on an actuation mechanism, may be categorized as active/powered and semi-active knee joints [12]. Powered transfemoral prosthesis (PTP) consists of actuators such as a DC motor coupled with a gear/screw-based transmission system, enabling amputees to perform more energy-demanding tasks (including stair/ramp negotiation) with less effort [13]. Although different research groups [14,15,16,17] have developed PTPs, Power Knee [18] by Ossur is the only PTP available for commercial use. The possible reasons include the weight of these devices, noise during operation, low battery time, complex architecture, non-intuitive user interface, and high development cost [19]. 

Contrary to PTPs, which provide assistive support to amputees, the variable damping prosthesis (VDP) only modulates the instantaneous resistance of the knee joint [20]. Possible options for varying knee joint resistance include pneumatic, hydraulic, and magnetorheological damper (MRD). Pneumatic dampers provide adequate damping in the swing phase for speed adaption. However, the stability of these dampers during the stance phase is insufficient [21]. Servo-controlled hydraulic dampers are also a common choice in commercially available VDPs; however, the disadvantage of hydraulic dampers is their complicated design, along with fluid leakage due to excessive pressure buildup [22]. MRD is a relatively new concept that takes advantage of smart fluids that instantaneously change from a free-flowing liquid to a semi-solid when exposed to a magnetic field generated by an electromagnet [23]. The yield strength of the semi-solid and, hence, the damping of MR fluid can be varied by changing the strength of the magnetic field [24]. The advantages of the MR-based VDP include the simple architecture and a wide range of damping, which can be adjusted by varying the average current passing through the electromagnet [25].

Herr and Wilkenfeld [11] pioneered introducing MRDs in VDPs. They developed a custom MR damper operating in shear mode followed by a finite state machine (FSM)-based architecture for varying the damping of knee joint in response to the data received from different sensors integrated locally in the prosthesis. The research ultimately resulted in the development of the Rheo Knee, commercialized by Ossur, providing numerous features to amputees, including forward/backward walking, sitting, standing, and running. After their study, other researchers also explored applications of MR dampers in TPs. Fu et al. [26] developed a custom shear mode MRD and integrated it into a TP, followed by a sliding-mode tracking controller to predict and control the swing angle of the shank. However, human subject testing was not performed; therefore, the significance of the developed prosthesis cannot be evaluated. The authors in [27] identified that, due to a fixed instantaneous center of rotation (ICR), uniaxial TPs cannot accurately replicate the motion of a normal human joint; therefore, the authors developed a polycentric custom MRD-based prosthetic knee. The developed device was controlled through a Proportional Derivative (PD)-based control system. However, similar to [26], prosthesis and control-system evaluation was not performed on human subjects. Park et al. [28] developed a hybrid knee joint consisting of a custom MR damper and an electronically commutated motor. A nonlinear PD-based controller was integrated with the computed torque method to predict and control the trajectory of the knee joint. Although the desired knee joint angle during walking is achieved, however, the system complexity increased significantly due to the integration of dual actuators. 

Although significant research has been performed on MRD-based VDPs, the application of these devices in full is yet to be explored in low-income/developing countries, mainly due to their whopping cost, ranging from USD 30,000–USD 60,000. Other factors contributing to the low acceptance rate of commercially available VDPs in developing countries include lack of technical facilities, expertise, and skills for providing sufficient training and lifelong care to amputees, along with the unavailability of services and locally manufactured components [29]. Consequently, the World Health Organization (WHO) reported that the affordability of functional prostheses is a major barrier to the rehabilitation of millions of amputees in developing countries [30,31,32]. 

The major factors contributing to the high cost of MRD-based VDPs include custom actuating and sensing mechanisms and artificial intelligence (AI)-based controllers for tracking knee joint position. To cater for these issues, few researchers have attempted to develop cost-effective VDPs. In their research work, Diaz et al. [19] proposed a polycentric four-bar-based knee joint employing MRD for variable damping. Although human trials were reported, however, they were limited to walking at a constant speed, which is not in line with the scope of MPKJs. Pandit et al. [33], in an attempt to reduce the cost of VDP, utilized an off-the-shelf MR damper and developed an insole-based sensing mechanism. Trials on a single amputee were conducted, and similar to [19], the behavior of the developed prosthesis at self-selected walking speed was not demonstrated. 

Furthermore, the insole-based sensing mechanism limits amputees to using instrumented shoes only, which may result in low acceptance of the developed device. The authors in [34] developed a TP consisting of an off-the-shelf MRD and finite state machine (FSM)-based control architecture for modulating the resistance of the knee joint. The sensing module consists of a single accelerometer and two force-sensitive resistors, one of which is mounted on the heel, whereas the other one is mounted on the toe of the foot. Although trials for walking on level ground were performed, the control architecture could differentiate between stance and swing phase only, resulting in an unnatural gait cycle. Furthermore, similar to [34], the amputee is forced to use an instrumented shoe, which affects the commercial viability of the device. 

From the discussion of the state-of-the-art, it can be summarized that there is still ample room to explore the development of cost-effective MRD-based VDP. Therefore, this study hypothesized that, without using any custom actuating and sensing mechanisms, an MRD-based VDP (i-Inspire Knee) could be developed to facilitate the amputee in performing ADLs. Finite element analysis (FEA) was performed to ensure that the i-Inspire Knee does not deform under different loading conditions when the amputee performs ADLs. Furthermore, the necessary sensing mechanisms are installed locally (within the chassis of the knee joint), thus eliminating the need for instrumented shoes. The gait phase identification is performed through sensor fusion of the data received from the knee angle sensor, Inertial Measurement Unit (IMU), and strain gauges. A three-level control architecture was developed to recognize amputee intent, switch between different phases of a given mode, and modulate damping in the selected phase. Trials were performed on a unilateral transfemoral amputee. During experimentation, kinetic and kinematic data were collected to assess the performance of i-Inspire Knee. The results suggested that the i-Inspire Knee can accurately recognize the intent of the amputee, assist him in walking at a self-selected speed, and control the knee flexion angle within limits to reduce the amputee’s stumble rate. Finally, the cost and feature comparison with the state-of-the-art suggests that i-Inspire can be a cost-effective alternative for amputees residing in developing countries.

## 2. Mechanical Design

**Figure 1:**
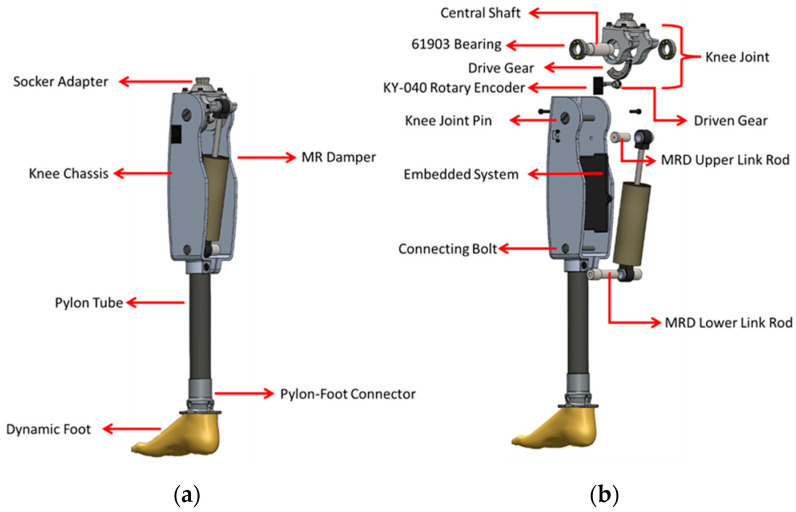
i-Inspire Knee (**a**). Three-dimensional model (**b**). Exploded view with component detailing.

The design of the i-Inspire Knee is based on the decision matrix presented in Table 1. The decision matrix comprises features adopted from [35] and specifications equivalent to commercially available VDPs. Based on these specifications, the developed 3D model of the i-Inspire Knee and its exploded view are shown in Figure 1. The design includes a knee joint, MRD, knee chassis (KC), pylon tube, and socket adapter. The KC houses an MRD, embedded system, sensing mechanisms, and battery to enhance the cosmetic appearance of the i-Inspire Knee and protect the components from mechanical shocks. The knee chassis is custom designed and manufactured through subtractive manufacturing from aluminum 6061, as it exhibits excellent machinability features and provides a sufficient strength-to-weight ratio. The knee chassis is connected to the knee joint through a central shaft supported by deep-groove ball bearings (SKF-61903) (SKF Co., Ltd., Lahore, Pakistan) secured inside the knee joint to ensure smooth and frictionless motion transfer from the amputee’s hip joint to the prosthesis. The knee joint is manufactured from aluminum 6061 and connected to a stainless-steel-304 ( Ottobock Co., Ltd., Duderstadt, Germany) socket adapter.

SS-304 was selected because the socket adapter bears the full body load of the amputee. This socket adapter is connected to the amputee’s stump through a custom-built socket and an off-the-shelf double adapter (Ottobock-4R72 Ottobock Co., Ltd., Duderstadt, Germany) to ensure distal and proximal angle alignment in the frontal and sagittal planes. Furthermore, the knee joint connects the MRD with the knee chassis through upper and lower link rods. Consequently, during flexion and extension of the knee joint, the piston reciprocates in the housing of the MRD. The bottom of the knee chassis is attached to a pylon tube, which, in turn, is connected to a dynamic foot. The knee chassis houses a gear-driven transmission system for measuring the knee joint angle. A rotary encode (KY-040 Shenzen Funkey Tech Co., Ltd., Shenzhen, China) is fixed to the outer wall of the knee chassis with a driven spur gear mounted at its shaft. The drive gear is a custom-cut spur gear that fits the outer profile of the knee joint. The drive and driven gears have a ratio of 1:2, which signifies that, apart from motion transmission, the knee angle sensor’s sensitivity was also enhanced.

**Figure 2:**
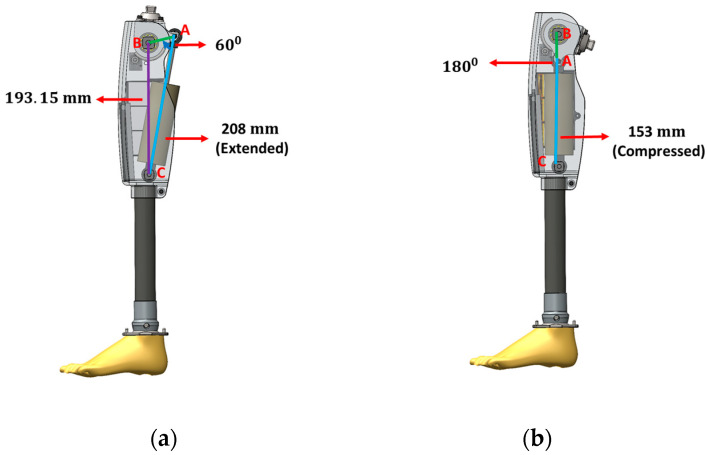
Kinematic model of MPK (**a**). Knee in straight position (**b**). Knee in full flexion.

The kinematics modelling was then performed to calculate the necessary link lengths for the efficient performance of i-Inspire Knee. Since the i-Inspire Knee is a single-axis knee joint accordingly, link lengths AB, BC, and AC are determined as shown in Figure 2, where we have the following:

- AB is the length from the knee joint axis to the upper busing axis of the MR damper;
- AC is the extended/retracted length of the MR damper;
- BC is the length from the knee joint axis to the lower bushing axis of the MR damper.

Since off-the-shelf MRD is used accordingly, the link length *AC* is 208 mm when the damper is in the extended position, as shown in Figure 2a, whereas it is 153 mm when the maximum knee flexion angle is achieved, as is evident in Figure 2b. Furthermore, *∠A* is kept at 60° when the MRD is in the extended position to achieve a maximum flexion angle of 120° when the MR damper is fully compressed, corresponding to *∠A* = 180°. In addition, the link length *BC* determines the overall build height of the prosthesis; therefore, to minimize the build height of i-Inspire Knee for targeting a larger population of transfemoral amputees, this length is fixed at 193 mm. Accordingly, the link length *BC* can be calculated using the law of cosines, as shown in Equation (1):

(1) $${AB}^{2}={AC}^{2}+BC^{2}-2\left(AC\right)\left(BC\right)cos(A)$$

By rearranging the equation and substituting necessary values when the MRD is in the extended position, the link length *BC* can be evaluated as 39.50 mm. 

The kinematic modelling was then followed by motion analysis of the i-Inspire Knee performed in SolidWorks to ensure that the desired knee flexion angle is achieved and there is no mechanical interference between the components. Finite element analysis (FEA) was performed in SolidWorks, as presented in Section 2, to ensure that the i-Inspire Knee can support the targeted weight without deforming.

## 3. Structural Analysis of i-Inspire Knee

The finite element analysis (FEA) was used to perform a structural analysis of the i-Inspire Knee. FEA can be defined as a numerical method for solving complex engineering problems to predict the behavior of the component(s) under given loading conditions. When fitted to an amputee, the TP becomes an integral part of his body, so performing an FEA is essential to ensure that the i-Inspire Knee can sustain different loading conditions without deforming while the amputee is performing ADLs. The next section discusses the boundary conditions used for performing the structural analysis of the i-Inspire Knee.

### 3.1. Boundary Conditions

**Figure 3:**
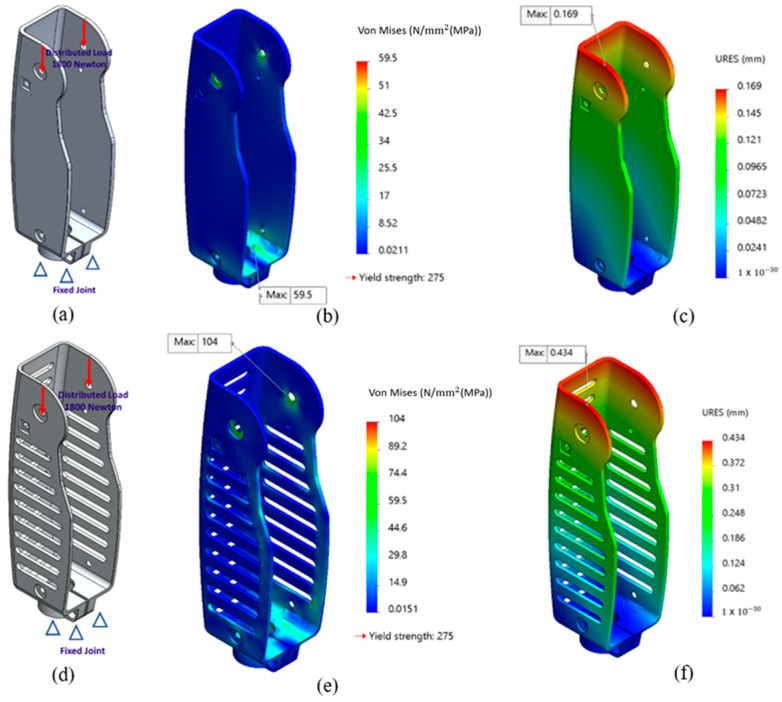
Finite element analysis of i-Inspire Knee. (**a**) Boundary conditions for the knee chassis, depicting 1800 N distributed load on the supports of knee joint and fixed bottom support. (**b**) Von Mises stresses of knee chassis. (**c**) Total deformation of knee chassis. (**d**) Boundary conditions for the updated knee chassis. (**e**) Von Mises stresses for the updated knee chassis. (**f**) Total deformation of the updated knee chassis.

Grabowski and Andrea [36] reported that a maximum of 140% of the body weight acts as a ground reaction force during the stance phase of the gait cycle when the amputee is walking on level ground with a speed of 1.75 m/s. Accordingly, for an amputee weighing 130 kg, a force of 1800 N was applied on the knee joint attachment points, and the bottom of the knee chassis is considered to be a fixed (or ground) joint, as shown in Figure 3. A high-quality mesh of the knee chassis was then obtained that consisted of 50,238 elements and 107,819 nodes.

### 3.2. Von Mises Stress and Total Deformation

After defining the boundary conditions, the von Mises stress determines whether a material will yield or fracture under specific loading conditions. Subsequently, the Von Mises yield criterion states that if the von Mises stress of a material under given loading conditions is equal to or greater than its yield limit, the material will permanently deform. In addition to the von Mises stress, total deformation was also calculated. Total deformation can be defined as a change in dimensions and, in certain cases, the shape of a body because of an applied external force. The deformation can be calculated in each dimension, such as the X, Y, and Z axes, whereas the total deformation computes the square root of the sum of the squares of the deformation in the individual dimensions.

### 3.3. Results

The selected material for the knee chassis is aluminum 6061, which has a yield strength of 275 MPa, Young’s modulus of 70 GPA, Poisson’s Ratio of 0.33, and density of 2700 kg/m^3^. Applying a force of 1800 N at the supports of the knee joint, as shown in Figure 3a, resulted in a maximum stress of 59.5 MPa (Figure 3b), maximum deformation of 0.169 mm (Figure 3c), and minimum factor of safety (FOS) of 4.5. Considering the large FOS, material removal and thickness reduction was considered to reduce the weight of the knee chassis. FEA was then repeated on the modified knee chassis, as shown in Figure 3d, and a minimum FOS of 2.7 was achieved. The maximum stress of 104 MPa (Figure 3e) was recorded at the top of the knee chassis; however, the stress is below the yield strength of aluminum 6061. Therefore, the knee joint will not plastically deform under extreme loading conditions. Furthermore, for the updated knee chassis the total defomation (Figure 3f) increased to 0.4 mm. However, ISO-10328 [37] presents that the knee joint is safe to use if, under extreme loading conditions, the total deformation does not exceed 5 mm. Therefore, it is concluded that a maximum deformation of 0.4 mm will not affect the normal operation of the prosthesis.

### 3.4. Fatigue Analysis

**Figure 4:**
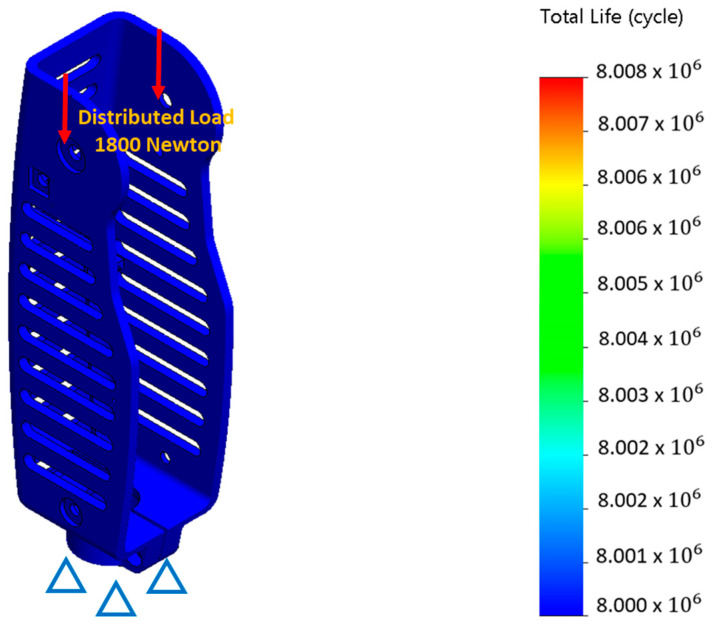
Fatigue analysis of i-Inspire Knee at a cyclic loading of 1800 N and a frequency of 1 Hz to predict total life cycle of knee chassis.

The fatigue analysis of the i-Inspire Knee followed the static structural analysis. Fatigue analysis was performed to assess the durability and life expectancy of the i-Inspire Knee under repetitive loading or cycling stresses. Accordingly, a load of 1800 N was applied to the knee joint attachment points at a frequency of 1 Hz. This frequency was selected because the stride time for a normal subject walking at a self-selected speed is approximately 1 s [38]. The fatigue analysis results are presented in Figure 4, where it is evident that the chassis can endure 8 million cycles under repetitive loading before encountering any structural collapse. Hence, the life expectancy of the i-Inspire Knee is greater than the standard 3 million cycles recommended by ISO-10328 for lower limb prosthesis. Therefore, it can be assumed that the developed prosthesis is durable and capable of withstanding different loading conditions that arise when the amputee performs ADLs such as walking and jogging.

## 4. Sensing System for i-Inspire Knee

The 3D modelling and structural analysis were followed by the development of a sensing system for the i-Inspire Knee. The sensing system for an MPKJ should be cost-effective and capable of accurate intent recognition, gait phase identification, and walking speed estimation to effectively modulate the damping of the actuator for assisting amputees in performing ADLs [39]. Accordingly, few authors reported low-cost force-sensitive resistors integrated into the shoe insole for gait phase identification [40,41,42,43]. However, with the said approach, the position of the knee joint cannot be reconstructed in 3D space, resulting in inaccurate amputee intent recognition. Furthermore, using custom footwear with instrumented insoles is impossible, making the technology commercially impracticable. To address this challenge, a few studies discussed the integration of multidimensional force sensors and custom IMUs in TF prostheses to improve the precision and accuracy of the sensing system [21]. However, the system complexity and cost of the prosthesis increased significantly. Consequently, the goal of this study was to develop a sensing system that is cost-effective, easy to implement, compatible with custom footwear, and exhibits excellent precision and accuracy. 

**Figure 5:**
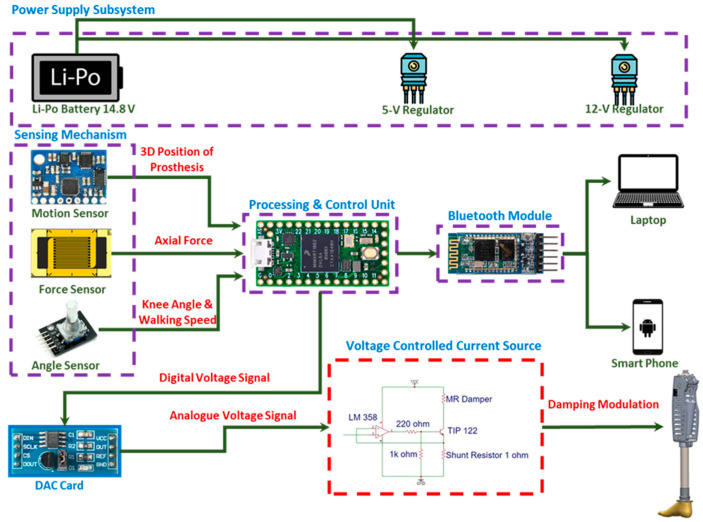
Block diagram comprising the sensing and control unit for the i-Inspire Knee.

**Figure 6:**
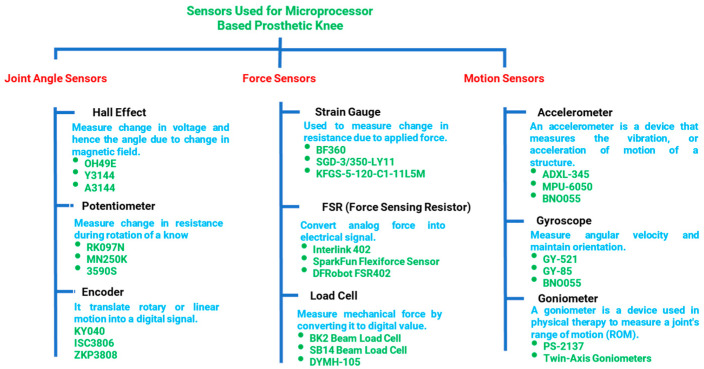
Possible sensors, along with their types and suggested models for integration in the knee joint, to recognize amputee intent and detect different phases of a given mode.

The overall architecture of the i-Inspire Knee, as summarized in Figure 5, consists of a power supply system, a sensing and processing unit, and an MRD for modulating the damping of the knee joint. The sensing mechanism consists of an IMU, force, and angle sensor. The available options for each sensor type are presented in Figure 6, whereas the functional parameter for individual sensors is discussed as follows:

- Angle sensor measures the inclination/angle of the knee joint and estimates the amputee’s walking speed.
- Force sensing is necessary for differentiating between the stance and swing phases of the gait cycle.
- Motion sensing assists in the reconstruction of the position of the prosthesis in 3D space for accurate intent recognition.

For measuring the knee angle, the Hall Effect sensor was not considered in this study, as it requires integrating a permanent magnet in the knee through which the voltage changes and, hence, the knee angle can be determined. This approach will increase the system’s complexity, and the permanent magnet may also interfere with the operations of the MR damper. Furthermore, the ratio between the drive and driven gear is (75:16); therefore, when the knee joint flexes to a maximum flexion angle of 120°, the drive gear and, hence, the angle sensor complete 1.56 revolutions. Therefore, the suitable solution is either a multi-turn potentiometer or an encoder that rotates continuously. The rotary encoder (KY-040) was preferred over the potentiometer, as it has a longer life expectancy and acceptable accuracy. The mechanical design section already discusses the integration and operating principle of rotary encoder-based knee angle sensing. 

Motion sensing assists in the reconstruction of the knee joint in space; therefore, the possible options include the IMU, gyroscope, and goniometer. For this purpose, a six-axis Inertial Measurement Unit (GY-521) integrated into the embedded system is preferred, as it consists of a three-axis accelerometer and a three-axis gyroscope. The data from the accelerometer and gyroscope were fused together, using a complementary filter, as discussed in [44]. Complementary filter was preferred over a Kalman filter, as it is computationally inexpensive and provides higher accuracy [45].

For force sensing, initially, the load cell (JHBM-H3) was considered by mounting it in between the knee chassis and pylon tube; however, during experimentation, the authors observed that the load cell in the said configuration is not capable of handling irregular loads during different phases of the gait cycle. Therefore, strain gauges (BF-350) were used in a standard Wheatstone bridge configuration for sensing ground reaction forces. Among four strain gauges, two each were placed on the opposite side walls of the knee chassis to measure the ground reaction force (GRF). 

To interface the BF-350 strain gauge sensor with the control unit, a 24-bit analog-to-digital converter (ADC) HX711 was used. The strain gauges were initially calibrated based on the weight of the amputee, and the calibration factor was then stored in the Electrically Erasable Programmable Read-Only Memory (EEPROM) of the microcontroller. Using a calibrated strain gauge and a reliable ADC, the controller could distinguish between the swing and stance phases of the gait cycle. The data of these three sensors (IMU, strain gauge, and angle sensor) were then fused by a processing and control unit to modulate the knee joint resistance according to the selected mode and phase of a given activity (see control architecture section). 

The sensing and control unit of i-Inspire Knee is powered by a rechargeable 11.1 V 800 mAh lithium polymer (LiPo) battery pack connected to a 12 V battery regulator for powering the MR damper and a 5 V regulator for powering the sensing and control unit. The battery is expected to require recharging after every 10,000 steps during ground-level walking. Considering an average stride length of 2.3 feet, the battery will power up the i-Inspire Knee and enable the amputee to walk 7 km at a nominal speed of 3.6 km/h. 

The processing and control unit consists of a Teensy 4.0 development board with a state-of-the-art 32-bit ARM Cortex M7 processing unit that is capable of operating the control loop at 600 MHz. In addition to collecting sensory data, Teensy performs secondary tasks such as data logging. For this purpose, the Bluetooth module (HC-05) is interfaced with Teensy. It can send the data to terminal devices via CoolTerm (Version 2.0.1), a freeware serial port terminal software. 

Teensy 4.0 also modulates the damping of MRD and, hence, the resistance of the knee joint to flexion/extension by varying the current passing through it. MRD is an actuator filled with magnetorheological (MR) fluid that responds to an applied magnetic field. It is typically composed of ferrous (iron) particles suspended in a liquid carrier, such as oil or water. When a magnetic field/current is applied to the fluid, the ferrous particles align themselves along the field lines, causing the fluid to exhibit a dramatic change in its viscosity. In simpler terms, the viscosity of the MR fluid can be controlled by adjusting the strength of the magnetic field, which is directly proportional to the current flowing through the MRD. When the magnetic field is increased, the fluid becomes more viscous, thus increasing its damping, or, alternatively, it can be said that the resistance of the piston to change in its position has increased. Similarly, when the magnetic field is decreased or removed, the fluid returns to its more liquid state, thus decreasing the resistance of the piston to change in its position. A LORD RD-8041-1 MR damper was used in this research with the specifications mentioned in Table 2. 

According to the working principle of the MR damper, the joint resistance controller consisted of a voltage-controlled current source circuit capable of converting input voltage changes to currents. A 10-bit digital-to-analog converter (TLC5615) was interfaced with a Teensy that controlled the duty cycle of the transistor (TIP41C) through Op-Amp (LM358). The Op-Amp was operated in a voltage follower configuration, ensuring that the voltage at the inverting and non-inverting terminal of the Op-Amp was equal. According to Ohm law, considering that the constant resistance, voltage, and current are directly proportional, greater input voltage will result in higher current in the MR damper. Consequently, by changing the input voltage, the current and damping of MRD can vary. The necessary electronics of the i-Inspire Knee were packed on a single board with dimensions of 80 mm × 20 mm that was integrated into the knee chassis, as already shown in Figure 1.

## 5. Control Architecture of i-Inspire Knee

**Figure 7:**
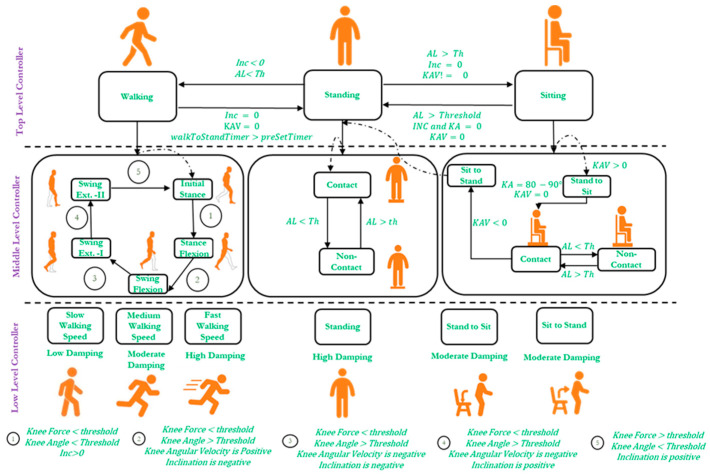
Three-layer finite state machine-based control architecture of i-Inspire Knee, consisting of a top mode selection layer, middle phase detection layer, and lower damping modulation layer.

The control architecture for i-Inspire Knee, as presented in Figure 7, consists of a three-level hierarchy, including a top-level controller, a middle-level controller, and a low-level controller. The top-level controller recognizes amputee intent and switches between different modes, whereas the middle-level controller detects different phases of a given mode. The low-level controller controls the MRD (knee joint) damping based on the phase information. The following conventions are adopted throughout the control architecture of the i-Inspire Knee:

- A positive slope (inclination angle) indicates the knee joint’s forward inclination.
- A negative slope (inclination angle) indicates the knee joint’s backward inclination.
- Zero slope (within ±3° of uncertainty) represents that the knee joint is perpendicular to the ground (as in the case of standing position)
- During flexion, with reference to the knee chassis, anti-clock wise rotation of the knee joint is observed, resulting in a positive angular velocity.
- During extension, with reference to the knee chassis, clocl-wise rotation of the knee joint is observed, resulting in a positive angular velocity.

### 5.1. Top-Level Controller

The top-level controller recognizes amputee intent and accordingly switches between the given modes. In this research, the i-Inspire Knee was programed with the modes commonly used by amputees for performing ADLs, such as ground-level walking, sitting, and standing. The switching between these modes depends on the sensory data and a set of timers. Beginning from the standing position, when the amputee takes a forward step with his sound leg, it shifts the amputee’s weight on the sound leg and backward inclination of the prosthesis. Consequently, when the inclination angle < 0 and axial force < threshold, the top-level controller switches the prosthesis from standing to walking mode.

Within the walking mode, when the IMU reports zero inclination for a given time, we have the following:

Inclination angle =0 and walkToStandTimer>preSetTimer and kneeAngularVelocity =0,

The controller activates standing mode. Similarly, if the amputee is in standing mode and the knee joint begins to flex/extend while IMU reports zero inclination, we have the following:

Inclination angle =0 and kneeAngularVelcoity! =0 and Axial Force > threshold

The controller switches between sitting and standing modes. 

### 5.2. Middle-Level Controller

Based on the decision of the top controller, the middle-level controller switches between different phases of a given mode, as shown in the middle-level controller depicted in Figure 7.

#### 5.2.1. Middle-Level Controller for Standing Mode

The standing mode consists of two distinctive phases, i.e., contact and non-contact. During the contact phase, the prosthetic knee is in contact with the ground and fully or partially supports the weight of the knee joint, whereas, during the non-contact phase, the amputee shifts his weight on the sound leg, and the prosthesis is unloaded. Consequently, when the top-level controller switches from walking to standing mode, the middle level assesses the axial load measured by the strain gauge. If the axial force > threshold, then the standing mode’s contact phase is activated, whereas, if the axial force < threshold, the controller switches to the non-contact phase. 

#### 5.2.2. Middle-Level Controller for Sitting Mode

The middle-level controller for sitting mode recognizes activities ranging from standing to sitting and sitting to standing and the amputee’s posture when sitting comfortably on a chair. As already discussed, the anti-clockwise rotation of the knee joint when the knee joint is perpendicular to the ground and the prosthesis is loaded with the amputee’s weight triggers activation of the sitting mode. Consequently, if the middle-level controller detects flexion of the knee joint such that  kneeAngularVelcotiy>0, a standing-to-sitting transition is detected. When the amputee completes the sitting process such that the kneeAngle ranges from 80° to 90° and the kneeAngularVelocity=0, the middle-level controller detects that the amputee is seated, and the comfort seating (contact) phase is activated. Afterwards, the amputee can freely extend or further flex the knee joint while remaining in a sitting position, resulting in switching to a non-contact phase. 

When the amputee intends to stand, the knee joint is loaded by the amputee’s weight. The knee angle recorded by the sensor is between 80° and 90°, followed by the extension of the knee joint such that  kneeAngularVelcotiy<0, then the controller switches from the standing to sitting phase. 

#### 5.2.3. Middle-Level Controller for Walking Mode

**Figure 8:**
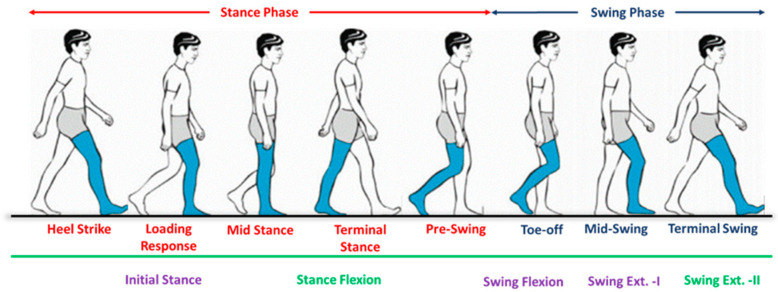
Visual representation of different phases of the human gait cycle.

During walking, a biologically realistic gait cycle is restored by the i-Inspire Knee; therefore, the middle-level walking controller is of key importance. The gait cycle can be broadly classified as the stance and swing phases [46]. During the stance phase, the foot, or some portion of it, is in contact with the ground, whereas, in the swing phase, the foot is lifted off the ground. As presented in Figure 8, a typical gait cycle begins with the heel strike, resulting in a flexion of 20° (stance flexion), followed by the loading response (flat foot). After the loading response, the knee joint fully extends (stance extension) until mid-stance and supports the amputee’s complete body weight. In between the mid-stance and pre-swing phase, when the toe of the foot is about to leave the ground, the knee joint again flexes, referred to as the pre-swing. The knee flexion continues as the foot leaves the ground until the maximum flexion angle (MFA) is achieved. The knee joint then extends (swing extension) and prepares for the next heel strike as one complete gait cycle is completed. The body then executes these phases repeatedly during walking. Instead of detecting phases such as heel strike or pre-swing, the middle-level walking controller predicts events such as stance flexion and swing extension. 

Recent studies [47,48,49] categorized gait phases into five distinct events, namely stance flexion (loading response), stance extension (mid-stance), terminal stance (pre-swing), swing flexion, and swing extension, with more emphasis on achieving a stance flexion of 10°–20° to absorb the energy resulting from the shock at the beginning of the step. However, the literature has reported that the user perceives this movement as a form of instability [50]. Furthermore, it was observed in a randomized crossover study that, with passive/microprocessor-based prosthesis, a very small flexion angle is achieved [51]. Therefore, during this research, the initial flexion during the loading response of the knee joint was not considered. Furthermore, swing extension has been considered a single event in a gait cycle; however, during the initial swing extension, the knee joint accelerates. Therefore, it is essential to decelerate the prosthesis during the late swing extension to prepare it for the next step. Consequently, the authors divided the swing extension into two distinct phases, i.e., swing extension (I) and swing extension (II). A detailed description of the gait phase’s events, along with necessary switching conditions, is discussed below:

The conditions for the detection of each gait event are summarized below:

- Initial stance: The initial stance is observed at the heel strike when the knee joint is inclined forward, and the prosthesis starts to bear the amputee load. Therefore, there is a positive inclination angle, knee loading less than the threshold, and a knee angle less than the preset threshold result in the detection of loading response. In this study, the initial stance was extended until the mid-stance phase, when it is assumed that the knee joint remains fully extended.
- Stance flexion: The knee joint flexes from mid-stance to pre-swing, resulting in a backward (negative) chassis inclination while the prosthesis still supports the body weight. Therefore, when the controller detects a negative inclination angle, knee loading greater than the threshold, and positive angular velocity, it activates the stance flexion phase of the gait cycle.
- Swing flexion: After the toe-off, the stance phase is completed, and the swing phase is initiated; hence, the prosthesis does not support body weight. However, the knee joint flexes until the maximum flexion angle (MFA) is achieved. Consequently, a negative knee inclination, knee loading less than the threshold, and positive angular velocity mark the beginning of swing flexion.
- Swing extension: Immediately after the attainment of the maximum flexion angle, the knee joint extends until the next heel strike. The swing extension can be identified when the knee loading is less than the threshold, and the angular velocity of the knee joint is negative. As discussed earlier, the swing extension is subdivided into two sub-phases, i.e., swing extension (I) and swing extension (II). Swing extension (I) begins after MFA. It lasts until mid-swing, during which the inclination of the prosthesis is negative, whereas, from mid-swing to the next heel strike, a positive inclination of the prosthesis is recorded, and, hence, the controller switches to swing extension (II).

### 5.3. Low-Level Controller

Based on the selected mode’s phase, the low-level controller modulates the knee joint’s damping. The regulation in the damping is guided by various mathematical relationships to compensate for changes in loading conditions and allow the amputee to confidently perform ADLs. 

#### 5.3.1. Low-Level Controller for Standing

As discussed in the middle-level controller for standing mode, there is a contact and non-contact phase. During the contact phase of the standing mode, the prosthesis provides the amputee with enough damping to prevent the stumbling of the amputee. The damping force during the weight-bearing phase is calculated according to Equation (2) [11]:

(2) $$F_{d}=1.5\times AL$$

where *AL* is the axial load measured by the strain gauges and is essentially the weight of the amputee.

During the non-weight-bearing phase, the amputee does not load the prosthesis with the body weight because he may be preparing to switch from standing to walking mode. Therefore, during the non-weight-bearing phase, the prosthesis does not provide any damping to the amputee. However, if the amputee switches from the non-contact phase to the contact phase, the damping is restored according to Equation (2).

#### 5.3.2. Low-Level Controller for Sitting

During the standing-to-sitting transition, the damping of the knee joint can either be kept constant or increased exponentially to provide support during knee flexion to cause the consistent loading of both legs so that the amputee can maintain a natural posture while sitting. Constant damping (depending on the amputee’s weight) is calculated based on Equation (3) [11] and is suitable for amputees who intend to sit at a fast speed while feeling little knee flexion resistance. Conversely, amputees who like to have more support or resistance to knee flexion require exponentially increasing damping, calculated through Equation (4) [52]. Equation (4) shows that the knee flexion angle (α) increases as the amputee performs a sitting activity. Accordingly, the damping of the knee joint is exponentially increased.

(3) $$F_{d}=1.2\times AL$$

(4) $$F_{d}=AL\times 0.08\alpha$$

Once the amputee is seated, he may remove the load on the prosthesis; hence, no damping is provided during the non-contact phase of the sitting mode to allow for free extension of the knee joint. The amputee initially loads the knee joint with the body weight, and the prosthesis enters the contact phase for standing. From there onward, either a constant or exponentially decreasing damping is provided by the prosthesis to assist the amputee in standing without buckling the knee joint. 

#### 5.3.3. Low-Level Controller for Walking

The damping of the developed prosthesis during walking is dependent on the gait phase identified by the middle-level controller. During the initial stance, the objective of the prosthesis is to support body weight; hence, high flexion damping/resistance is provided to prevent the buckling of the knee joint. Throughout stance flexion and swing flexion, the flexion damping is kept at a minimum so that the amputee can easily flex the i-Inspire Knee and achieve the desired MFA. During swing extension (I), the hip flexion is created by hip flexor muscles to produce forward swing movement and, hence, acceleration in the knee joint; therefore, extension damping is kept at a minimum value to facilitate straightening of the prosthesis. However, during swing extension (II), it is essential to safely decelerate the prosthesis to avoid the sensation of uncontrolled extension in the amputee. Therefore, during swing extension (II), sufficient damping is provided by the low-level controller for the deceleration of the prosthesis. 

In addition to damping modulation during different phases of the gait cycle, the low-level walking controller also performs speed-adaptive control (SAC), enabling the amputee to walk at self-selected speeds (SSSs). The walking speed of the amputee can be estimated by calculating the contact time of the foot (stance phase) with the ground, as the literature suggests that there is an inverse relationship between contact time and walking speed [53]. To make SAC computationally inexpensive, the walking speed of the amputee was categorized into three distinct classes, i.e., slow, medium, and fast walking. Unlike previous studies [19,21,34] in which SAC was only realized through the control of MFA by modulating damping in swing flexion (I), the proposed study, in addition to swing flexion (I), varied the damping of the stance flexion and swing extension (II) as well because the damping in these phases is largely dependent on the walking speed of the amputee.

For this purpose, the amputee walked at slow, medium, and fast walking speeds in the presence of a trained prosthetist. The damping parameters for stance flexion (I), swing flexion, and swing extension (II) were individually tuned to realize a biological natural gait cycle. The tuned damping values for individual phases at different walking speeds are then stored in the controller’s non-volatile memory. Afterward, the controller computes the contact time, searches in non-volatile memory for damping values, and activates the desired damping for that walking speed. 

Furthermore, a target was defined for swing flexion to modulate damping at different walking speeds in real time. The target for swing flexion was to achieve an MFA of 45°–65°. If the prosthesis reaches an MFA of less than 45° at any walking speed, the damping in the coming steps is reduced by a small increment until the flexion angle is within the set target again. Similarly, if MFA is greater than 65°, it not only results in excessive heel elevation but also results in delayed extension, resulting in an unnatural gait cycle [54]. Therefore, the damping is increased in increments to ensure that MFA is achieved within limits.

## 6. Evaluation

**Figure 9:**
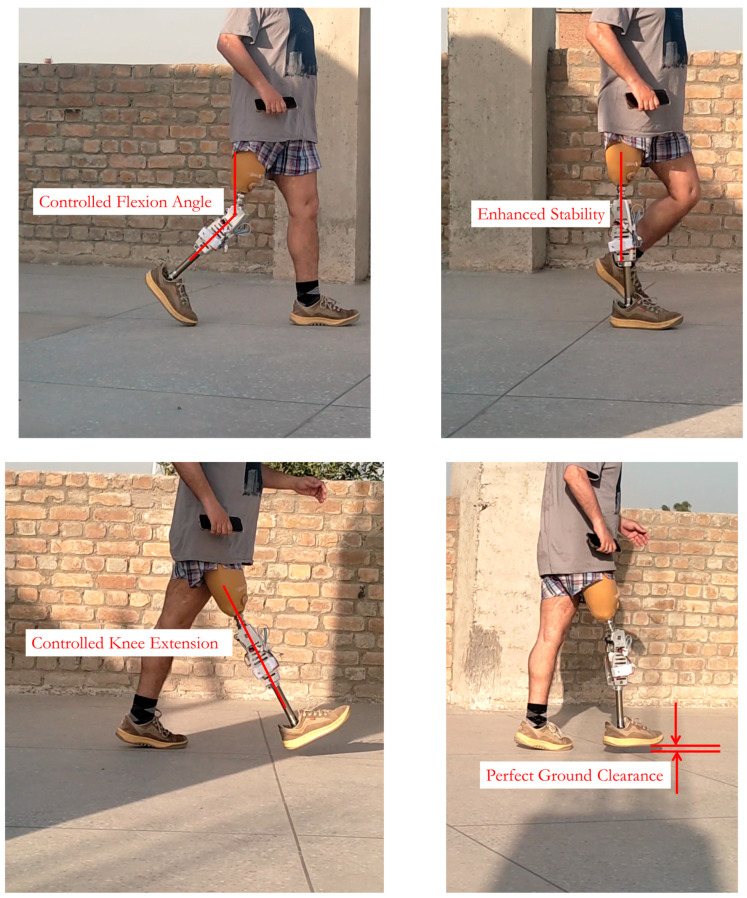
i-Inspire Knee evaluated on a transfemoral amputee and depicting its features.

The clinical evaluation of the i-Inspire Knee was performed on a 38-year-old unilateral transfemoral amputee, previously using a mechanical knee joint for the last five years. Figure 9 presents an i-Inspire Knee fitted to the amputee for evaluation purposes. Table 3 presents the basic data of the amputee, along with the details of different components integrated with the i-Inspire Knee to connect it with the amputee’s stump. The subject was a right-side amputee, and the AMP score was assessed to be above the K3 level. A custom socket of quadrilateral type was designed for the amputee by a licensed prosthetist. Through a double adapter, the knee axis of the amputated and sound side was matched.

Similarly, the length of the pylon tube was adjusted according to the height of the amputee, followed by dynamic alignment of the prosthesis. The amputee was comfortable with the socket and did not report any phantom limb pain, stump pain, and/or skin irritation. Prior to testing, the subject was briefed about testing protocols, and, accordingly, informed written consent was obtained from him. The ethical committee of the University of Engineering and Technology, Peshawar, approved the evaluation protocols.

## 7. Results

The proposed sensing and control architecture is implemented in real-time on the subject to assess the effectiveness of top-, middle-, and low-level controllers by evaluating intent recognition, gait phase identification, walking at SSS, and damping modulation during different modes and subsequent phases.

### 7.1. Evaluation of Top-Level Controller

**Figure 10:**
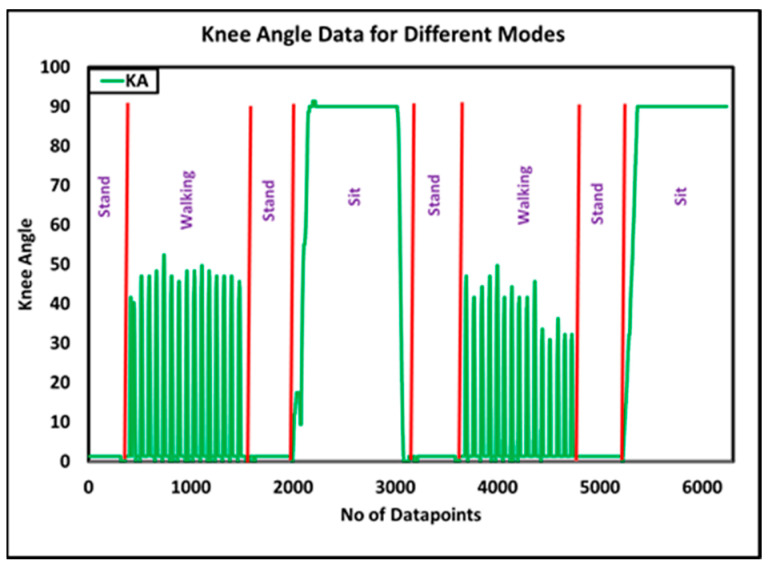
Knee angle data of i-Inspire during the evaluation of top-level controller.

Three trials were conducted to assess the performance of the top-level controller in recognizing amputee intent. The trials were performed inside a hallway, where chairs with armrests were placed at the starting and ending points. The back of the chair rested against a pillar to avoid accidents while the amputee was sitting on the chair. Each trial began with the amputee standing for 5 s, followed by a 25 m walk at SSS till the end of the walkway, where, again, the amputee maintained a standing posture for 5 s before sitting on the chair. The amputee then observed a rest period of 30 s, followed by standing for 5 s and walking back to the start of the walkway, where he stood for 5 s before sitting on the chair. During three trials, 24 mode transitions were observed, and all 24 were correctly identified by the controller without input from the amputee. Figure 10 presents the corresponding knee angle data and the decision of the top-level controller for one such trial.

### 7.2. Evaluation of Middle-Level Walking Controller

**Figure 11:**
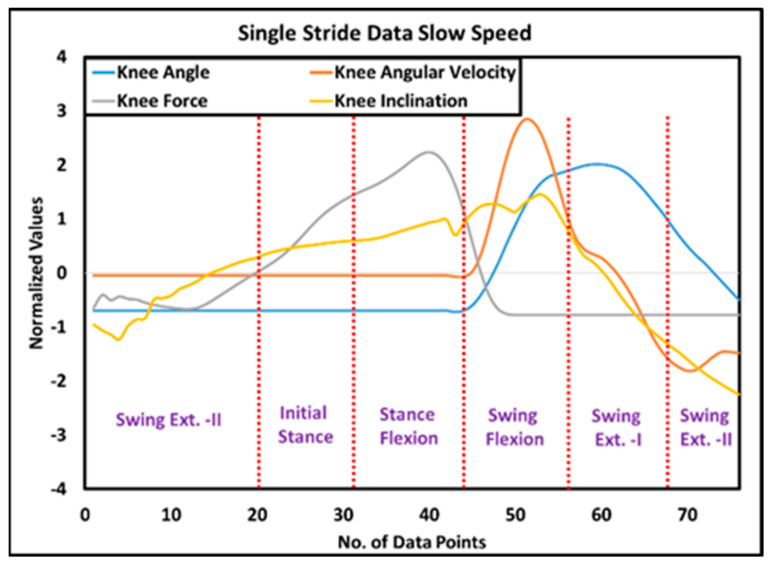
Data of knee angle recorded by rotary encoder, knee force measured by strain gauges, knee inclination deduced from Inertial Measurement Unit, and knee angular velocity obtained by differentiation of knee angle data for a typical gait cycle.

**Figure 12:**
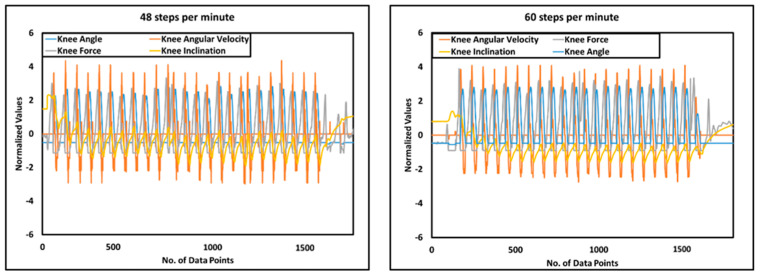
Sensor data for walking at slow and medium speed.

Among different middle-level controllers, walking is of key importance, as it detects different phases of the gait cycle for restoring the mobility of the amputee. The sensory data for a typical gait cycle are presented in Figure 11, whereas, data of knee angle, knee force, knee angular velocity and knee inclincation for 48 steps/min. and 60 steps/min. is recorded and plotted in Figure 12.

Since off-the-shelf cost-effective sensors were used in i-Inspire Knee, the sensing system’s accuracy and repeatability were evaluated via a statistical analysis. For this purpose, six trials were conducted at two different walking speeds. During each trial, the amputee maintained a standing posture for 5 s, followed by walking across the hallway. At the end, the amputee remained standing for 5 s. For all trials, the data of the sensors were collected and recorded. Prior to statistical analysis, three steps from the beginning and end of the walking mode were excluded, as they may be outliers or extreme values that do not represent the typical behavior of sensors during stable walking. Z-score normalization was applied to the data to standardize the measurements across different speeds.

Liu et al. [55] reported using the intraclass correlation coefficient (ICC) in an intent recognition approach for an above-knee prosthesis to assess the consistency of the sensor data between different walking speeds and terrains, helping to improve the recognition accuracy. Therefore, for every trial, the coefficient of correlation (COCR), and coefficient of covariance (COCV) for the selected number of strides were calculated for knee angle, IMU, and strain gauge, using Equations (5) and (6), respectively. Once the COCR and COCV were computed among all the strides, their mean and standard deviation were calculated to summarize the data, as presented in Table 4.

(5) $$Cov(X,\;Y)=\Sigma \;[(Xi-\;X^{¯})\;(Yi-\bar{Y})]\;/\;(n-1)$$

(6) $$r=(\Sigma \;[(Xi-X^{¯})\;(Yi-\bar{Y})])\;/\;(n-1(SD(X)\;*\;SD(Y)))$$

(7) z=((x−μ))/σ

where *μ* is the mean of the data, and *σ* is the standard deviation.

The lowest mean COCR was recorded between knee inclinations data when the amputee was walking at a medium speed; however, the recorded value of 0.850 still indicates a strong positive relationship between knee inclinations sensed by IMU across different strides. All other mean COCRs are greater than 0.850. Hence, it is safe to assume that sensors provide repeatable, consistent, and reliable data to the controller for mode detection and intent recognition.

### 7.3. Evaluation of Low-Level Current Controller

**Figure 13:**
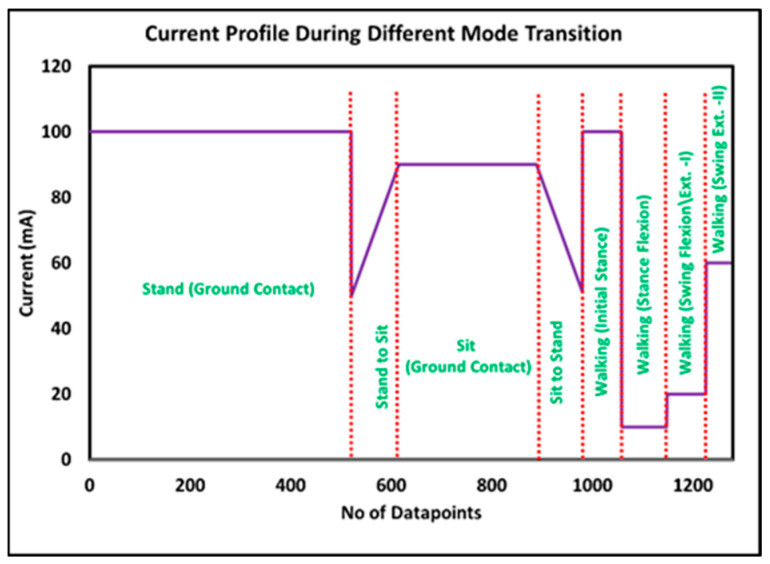
Damping profile for different activities.

The low-level controller modulates the damping of the developed prosthetic Knee according to the specific phase (detected by the middle-level controller) of a given mode to enhance the stability and improve the performance of prosthetic devices. Figure 13 presents the damping profile for when the amputee initially stood and transitioned to sitting mode. Afterward, the standing mode was again activated, followed by walking on level ground. The standing mode is the default mode of the prosthetic knee, where the user is stationary, and the knee is fixed in place. In this mode, the damping is set to a fixed value of 100 mA to ensure that the knee remains stable and bears the weight of the amputee. During the transition from standing to sitting, the damping is gradually increased to slow down the movement and prevent the user from falling forward. Similarly, during the transition from sitting to standing, the damping is gradually decreased to allow the knee to straighten smoothly. During walking, the damping in the initial stance is set at 100 mA, which is the period when the foot is in contact with the ground. This helps to provide stability and support during weight bearing. The damping is reduced to 10 mA in stance flexion to provide smooth foot liftoff. However, during the swing phase, the damping is adaptive when the foot is lifted off the ground. It adjusts itself according to the speed of the prosthetic knee. This allows the knee to move more freely and smoothly during the swing phase, which is important for an efficient and natural-looking gait. In swing extension II, the damping is set to 60 mA to provide smooth ground contact and some flexion, as whole-body weight is about to be transferred. The current values were selected based on the study of Jamadar et al. [56]. The authors present a mathematical model for magnetorheological dampers based on their equivalent damping and characterize the behavior of a commercially available magnetorheological damper, using the Bingham and equivalent damping models.

### 7.4. Evaluation of Speed-Adaptive Control Algorithm

**Figure 14:**
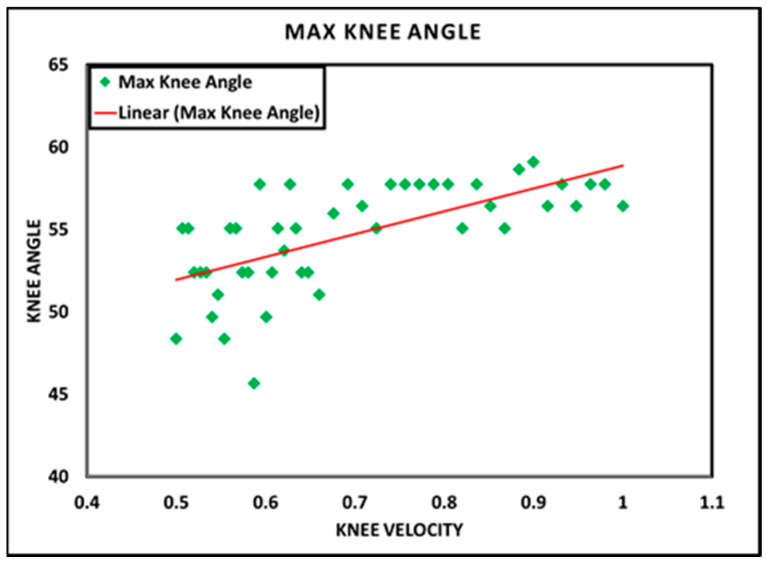
Linear relationship between knee angle and knee angular velocity.

As discussed previously, knee angle data (specifically MFA) are a benchmark parameter for evaluating the performance of SAC [11]. Accordingly, several trials were conducted during which the amputee walked at a self-selected speed, and, subsequently, knee angle data were recorded and are plotted in Figure 14. From the figure, it is evident that the damping of the knee joint is modulated to achieve a targeted MFA between 50° and 60°.

### 7.5. Comparison with State-of-the-Art Commercial Prosthesis

The comparison of the i-Inspire Knee with commercially available prosthesis is presented in Table 5. In real-time, the knee joint uses off-the-shelf sensors and an MR damper to modulate the knee joint resistance. Although i-Inspire knee offers standing, sitting, and walking modes only, since the joint is equipped with necessary sensors and controller, other features, such as ramp-down, stair descent, and knee-locking can be integrated into the i-Inspire Knee technology without additional hardware cost. The weight of the i-Inspire Knee is 1850 g, nearly 200 g more than the Rheo knee; however, by updating the material of the knee chassis from aluminum to carbon fiber, a 185 g weight reduction can be expected in future versions.

The cost of i-Inspire Knee is expected to be around USD 1600, as summarized in Table 6. The cost estimation consists of the equipment cost for electronics components, whereas the cost of the mechanical components is inclusive of the material and manufacturing cost. The manufacturing cost is calculated on the basis of standard rates applicable in the region where the research is performed. In addition to components’ cost, the HR cost, consisting of a mechanical design engineer and electronics engineer, is also considered. In total, 15% of the components’ cost is included, and institutional overheads are added to the cost of the developed prosthesis. Finally, a standard 25% profit is added to the total cost to calculate the selling price of i-Inspire Knee.

## 8. Conclusions

Microprocessor-based transfemoral prostheses are gaining importance to restore the gait cycle of amputees and assist them in becoming active citizens to contribute toward socioeconomic growth. However, the excessive cost of these devices is a restricting factor for most of the amputees in acquiring these devices. 

This study presents the development of a cost-effective magnetorheological-based transfemoral prosthesis (i-Inspire Knee). I-Inspire utilizes off-the-shelf sensing and actuating mechanisms and is equipped with all necessary features available in state-of-the-art microprocessor-based prostheses to perform activities of daily living. The mechanical stability of the i-Inspire Knee is ensured through statical finite element analysis and fatigue analysis. The results show that i-Inspire has a life cycle of 8 million steps, per the ISO-10328 standard. Considering an average of 4000 steps of amputee per day, this corresponds to a life of about 5.5 years. 

A state-of-the-art three-layer control architecture was developed for the i-Inspire Knee, making it capable of automatically recognizing different modes, including walking, sitting, standing, etc. For different modes, the i-Inspire Knee can detect phases and modulate the damping of the knee joint in real time to assist amputees in activities of daily living and walking at self-selected speed. The i-Inspire Knee can achieve a flexion angle of up to 120°, facilitating amputees in ascending/descending stairs and walking on ramps. 

The performance of the i-Inspire Knee is evaluated on a transfemoral amputee. Since off-the-shelf sensors are used, different tests were performed accordingly to test the repeatability of sensors. For this purpose, coefficients of correlation and coefficients of covariance were computed. These coefficients present a strong relationship between the sensor data among various trials. Thus, it can be concluded that sensors provide repeatable, consistent, and reliable data to the controller for mode detection and intent recognition. Finally, the comparison of the i-Inspire Knee with the state-of-the-art microprocessor-based prosthesis was conducted, and a detailed cost-breakdown of i-Inspire Knee is presented in this paper, from which it can be concluded that i-Inspire Knee has a cost of around USD 1500 and provides features comparable to state-of-the-art microprocessor-based prosthesis. 

## Figures and Tables

**Table 1:** Decision matrix consisting of features and corresponding specifications for the development of i-Inspire Knee.

Sr. #	Feature	Specification
1	Weight-bearing capacity	Support patient weight up to 130 kg
2	Max. knee flexion angle	120°
3	TP weight	1200–1800 g
4	TP cost	Less than 1500 USD
5	Walking controller	FSM-based variable damping
6	Available modes	Walking, sitting, standing, knee locking
7	Self-selected walking speed	Control knee flexion at different walking speeds within a range of 55–65°

**Table 2:** LORD RD-8041-1 MR damper’s (short stroke) specification.

Parameter	Value
Stroke, mm (in)	55 (2.17)
Extended length, mm (in)	208 (8.2)
Body diameter, mm (in)	42.1 (1.66) max
Shaft diameter, mm (in)	10 (0.39)
Tensile strength, N (lbf)	8896 (2000) max.
Damper forces, N (lbf)	
Peak to peak	
5 cm/s @ 1 A	>2447 (>550)
20 cm/s @ 0 A	<667 (<150)
Operating temperature, °C (°F)	71 (160) max.

**Table 3:** Data of the amputee, along with the components used in the prosthesis.

Sr. #	Parameter	Value
1	Height (m)	1.75
2	Weight (kg)	95
3	Age (years)	38
4	Amputation side	Right
5	Stump length (cm)	30
6	Prosthetic foot	Flex foot
7	Socket design	Quadrilateral

**Table 4:** Statistical parameters for slow and medium walking.

Sr. #	Statistical Analysis	Slow Knee Angle	Slow Knee Force	Slow Knee Inclination	Medium Knee Angle	Medium Knee Force	Medium Knee Inclination
1	Total strides	26					
2	Stable strides	20					
3	Mean coefficient of correlation	0.982	0.942	0.825	0.946	0.928	0.758
4	SD coefficient of correlation	0.0142	2.53	2.51	0.050	6.73	6.049
5	Mean coefficient of covariance	450.22	3401.44	402.97	456.56	1992.60	897.88
6	SD coefficient of covariance	29.48	160.28	22.41	54.58	150.37	76.50
7	Average step time	1.2 s (48 steps/min)			1.02 s (60 steps/min)		
8	Average speed	0.66 m/s			0.99 m/s		

**Table 5:** Comparison of the developed prosthetic knee with other knees.

Sr. #	Technical Features	Orion [57,58]	Pile [59,60]	Rheo [61,62]	Genium [63,64]	i-Inspire
1	Type of actuator	Hydraulic and pneumatic cylinder	Hydraulic cylinder	MR damping	Hydraulic cylinder	MR damping
2	Sensors	Piston stroke sensor; FSR; IMU	Inductive sensor; Strain gauges; load cell	Customized knee angle sensor; load cell; IMU	Knee angle sensor; load sensor; IMU	Knee angle sensor; strain Gauge; IMU
3	Walking controller	FSM variable damping	Two-point variable damping	FSM variable damping	FSM variable damping	FSM variable damping
4	Intent recognition	HRB approach, using timers, force sensor, and stroke sensor	HRB, using load cells and timers	HRB based on timers, load cell, and knee angle data	HRB based on sensory data	HRB, using IMU, strain gauge, and knee angle sensor
5	Modes	Sitting; standing; walking; RD; RA; SD; C; SR; K	Sitting; walking; standing; RD; SA; SR; UMI; C	Sitting; standing; walking; KL; RA; RD; K	Sitting; standing; walking; RA; RD; SA; SD	Sitting; standing; walking; knee Locking; SR (in-progress)
6	Cost	USD 30,000	USD 30,000	USD 40,000	USD 60,000	USD 1594

**Table 6:** Cost breakdown of i-Inspire Knee, listing electrical components’ cost, material and machining cost, HR cost, institutional overheads, and profit and selling price of i-Inspire Knee.

Sr. #		Component	Price in USD	Total Price
1	Electronics components	Voltage regulator—7812 and 7805	0.35	572.54
2		Teensy 4.0	35.33	
3		MR damper	511	
4		IMU MPU6050	1.24	
5		Strain gauge amplifier HX711	0.39	
6		DAC TLC5615	2.65	
7		Knee encoder (KY-040)	0.39	
8		Battery 14 V 2200 mAH	10.60	
9		Strain gauges—BF350	3.53	
10		LM358 with accessories	1.77	
11		TIP-122 transistor	0.35	
12		Bluetooth HC-05	3.53	
13		Wires, connectors	1.41	
14	Mechanical components	Socket adaptor	10.00	101.75
15		Central shaft	5.00	
16		Knee joint	16.50	
17		Ball bearing (SKF-61903)	13.50	
18		Knee chassis	46.75	
19		Miscellaneous, including connecting bolts, drive gear, and driven gear	10.00	
Cost of component				674.29
19	HR cost			500.00
20	Overhead			101.15
Cost price				1275.44
21	Profit @ 25%			318.86
Selling price				1594.30

## Data Availability

Data available on request due to restrictions.
